# Assessment of the State of Knowledge about HPV Infection and HPV Vaccination among Polish Resident Doctors

**DOI:** 10.3390/ijerph18020551

**Published:** 2021-01-11

**Authors:** Katarzyna Smolarczyk, Wojciech Pieta, Slawomir Majewski

**Affiliations:** 1 Department of Dermatology and Venereology, Medical University of Warsaw, 02-091 Warsaw, Poland; slawomir.majewski@wum.edu.pl; 2 Department of Gynaecological Endocrinology, Medical University of Warsaw, 02-091 Warsaw, Poland; wpieta2@wp.pl

**Keywords:** HPV, HPV vaccination, human papillomavirus

## Abstract

HPV (human papillomavirus) vaccinations have been introduced into the population of many countries through vaccination programs, although their acceptance varies from country to country, largely dependent on the state of knowledge about diseases caused by genital HPV types as well as cultural, social, and religious factors. The aim of the study was to analyze the state of knowledge about HPV and HPV vaccines among doctors during their specialization in gynecology and obstetrics, dermatology and venereology, and pediatrics. Another objective of the study was to analyze the impact of the state of knowledge about HPV vaccination on their attitude to primary prevention, i.e., vaccinations. A questionnaire was used to collect the data and 639 doctors took part in the study. The analysis was carried out mainly using descriptive statistical methods. In Poland, doctors’ knowledge about HPV is low, independent of gender, age, and subject of specialization. Doctors’ knowledge about the HPV vaccine is very low and independent of sex, age, and subject of specialization. However, doctors’ knowledge about HPV and the HPV vaccine influences the attitude to HPV vaccination and does not affect pro-active behaviors.

## 1. Introduction

### 1.1. Spectrum of HPV Lesions

HPV infection can be of two types: symptomatic and asymptomatic. Asymptomatic infections have been poorly studied. Approximately 15–20% of asymptomatic people can be diagnosed with features of HPV infection by the use of molecular tests [1].

Benign HPV lesions are common warts, genital warts, and recurrent respiratory papillomatosis (RRP). Genital types of HPV may also predispose to the development of precancerous lesions in the anogenital area, i.e., CIN (cervical intraepithelial neoplasia), VIN (vulval intraepithelial neoplasia), VaIN (vaginal intraepithelial neoplasia), AIN (anal intraepithelial neoplasia), and PIN (penile intraepithelial neoplasia).

Cancers proven to be related to HPV include cancer of the cervix, vulva, vagina, penis, anus, pharynx, and mouth.

In 2008, Harald zur Hausen was awarded the Nobel Prize in Physiology or Medicine for his discoveries of the role of HPV in the pathogenesis of cervical cancer. Cervical cancer is the fourth most common cancer affecting women and the seventh most common in the general population (2012) [2]. It has been estimated that there were 528,000 new cases in the world in 2012.

The incidence of cervical cancer in Poland has been falling in recent years. This success is attributed to the cervical cancer prevention program launched in 2006. This program is aimed at women between the ages of 25 and 59 who have not had a Pap smear test in the last three years. The incidence of cervical cancer fell from 11.5 per 100,000 women in 2005 to 10.3 per 100,000 women in 2010 [3]. The mortality rate has also decreased over the same period but remains very high. In 2015, according to the National Cancer Registry, there were 2723 new cases of cervical cancer and 1585 women died. In 2014, cervical cancer accounted for 3.6% of all cancer cases among women.

A large meta-analysis of 11 studies found HPV in 95% of cervical cancers. Oncogenic types (16, 18, 31, 33, 35, 39, 45, 51, 52, 56, 58, 59, 68, 73, 82) and probable oncogenic types (26, 53, 66) were selected [4]. The most common oncogenic type in cervical cancer is type 16 (53% of cancers plus CIN1–3) [5].

Every year in Poland, there are about 250 new cases of penile cancer, 250–300 new cases of rectal cancer, and about 500 new cases of vulvar cancer [6].

### 1.2. HPV Infection Prophylaxis

Prophylaxis can be divided into primary prevention, secondary prevention, and tertiary prevention.

#### 1.2.1. Primary Prophylaxis

Primary prophylaxis of HPV infections includes various types of procedures aimed at preventing the emergence and spread of infection. Primary prevention, in addition to vaccination, also includes educational activities aimed at eliminating or reducing the risk factors of HPV infection.

#### 1.2.2. Secondary Prevention: Pap Smear, Colposcopy, HPV Test

A Pap smear is a screening test for the prevention of cervical cancer. In Poland, a national screening program for cervical cancer was introduced in 2006. This program covers preventive examinations for women 25–59 years of age who are offered smear tests by invitation as well as training doctors and midwives.

A cervical smear test makes it possible to recognize pre-cancerous changes, as well as the duration of the menstrual cycle and the term of ovulation. A Pap smear is the first step in cervical cancer screening and further management depends on its result and follow-up tests.

Despite invitations to preventive examinations, cytology is poorly accepted in Poland. Women are ashamed of taking this test or are afraid of taking it for fear of the results of the diagnosis. The Pap smear also has its limitations in the form of the subjectivity of the assessment of the material by the assessor and the experience of the doctor collecting the material.

In Poland, colposcopy is not a screening test, but a test that verifies abnormal cytological results. This procedure, performed using a colposcope, makes it possible to indicate precisely the place from which—in the event of an abnormality—a biopsy should be taken. In Germany, colposcopy is a test that complements the Pap smear test. In Poland, it is more difficult to access due to the lack of support from the National Health Fund, the time-consuming procedure (compared to cytology), and the lack of qualified personnel. The varieties of colposcopy are penisoscopy, anoscopy, and vulvoscopy.

HPV detection tests are additional tests supporting the management of neoplasia. In Poland, they are available, but they are rarely performed due to the lack of reimbursements from the National Health Fund. 

#### 1.2.3. Barriers and Uptake: Barriers to the Implementation of HPV Vaccination

Many factors influence the level of inoculation and reception of the vaccine. The main ones include: lack of knowledge about HPV infections and vaccine, as well as motivational obstacles (“bad attitude” to vaccination in anti-vaccine environments, no recommendation from the National Health Provider or doctors, lack of support and conversations with parents about sexuality). 

Logistic barriers include: vaccination availability, the price of the vaccine, and the need to repeat vaccination (compliance). Vaccine myths—mistaken beliefs include sexual promiscuity, and negative information about the vaccine in the media (ineffective, not well researched, dangerous).

#### 1.2.4. Doctors’ Attitude towards HPV and HPV Vaccination

1. Doctors’ recommendations play key roles in the prevention of cervical cancer. A lack of recommendations from the NHP (National Health Provider; this is the NFZ in Poland) and doctors is a well-researched topic in previous studies. People who received a positive recommendation from the NHP and/or their doctor were more likely to get vaccinated or vaccinate their children.
2. It has also been previously studied that parents who had been strongly advised by healthcare providers to vaccinate their children had higher acceptance rates to immunization for their girls and boys and were more hesitant to postpone the immunization appointment and had fewer concerns about vaccines and were less likely to refuse vaccination [7].
3. In previously conducted studies, the knowledge of health professionals varies depending on age [8], region [9], level of education [9], and the number of years since training [10]. The relationship between HPV knowledge and pro-vaccine behavior is a problem that has already been discussed. Although in a study by Nidhi Jan et al., HPV-related knowledge was not significantly associated with the provision of counseling messages (STD, HPV, or cervical cancer prevention messages) [11], knowledge about HPV and HPV vaccination has been discussed as one of the factors associated with a willingness to prescribe HPV vaccines, which is a pro-vaccine behavior.

### 1.3. Aims of the Study

Poland still remains high in the rankings for the incidence and mortality of cervical cancer. Despite this, there are no reliable studies defining the state of knowledge about HPV infections, primary and secondary prevention, and the impact of the state of knowledge about the attitude towards vaccination. This study, the first of its kind undertaken in Poland, undertook an analysis to determine the barriers to vaccination acceptance and factors to determine vaccination in our country.

The aim of the work was to make an assessment of doctors’ knowledge about HPV infections, an assessment of doctors’ knowledge about HPV vaccines, and an assessment of the impact of doctors’ knowledge about HPV infection and HPV vaccines on their attitude to primary prevention, i.e., vaccinations.

## 2. Materials and Methods 

### 2.1. Design of the Study

An observational cross-sectional descriptive study was carried out.

All subjects gave their informed consent for inclusion before they participated in the study. The study was conducted in accordance with the Declaration of Helsinki, and the protocol was approved by the Ethics Committee of Medical University of Warsaw.

### 2.2. Data Collection

Data was collected using paper forms during obligatory study courses during residency. 

A researcher who monitored the conducted study and helped with technical problems was present to ensure that only residents were involved in the study.

The survey was conducted in the largest cities in 2018 throughout Poland.

No doctor refused to fill in the questionnaire, and those who did not complete it at a given course stated that they had completed one on a previous course. The study was completed when data from a minimum of 200 doctors of a given specialization were obtained.

### 2.3. Statistical Analysis

The analysis was conducted mainly with the use of descriptive statistics. The results are presented in the form of frequency tables and cross tables. Statistical inference was performed using the Chi-squared test or, in the case of low frequencies of the analyzed features, Fisher’s exact test.

The Fisher’s exact test is used in the case of samples that are too small—when the observed values calculated with the Chi-squared test are below 5. The calculations were performed using the statistics program R 3.5.1. All tests were performed at a significance level *p* = 0.05.

The responses of the doctors were presented depending on their age, sex, and size of the place of residence. Doctors’ knowledge about vaccination was also analyzed. The doctors’ responses to the questions related to their knowledge about vaccinations were classified as correct or incorrect, and each question was assigned a percentage. The attitudes of doctors towards the vaccination were then analyzed according to the results obtained.

### 2.4. Group Size

A goal of the study was to include as many participants as possible. At the initial stage, no formal calculation of sample size was carried out. The final number of 639 doctors ensured a precision (measured at half the length of the 95% confidence interval) of 4 percentage points for assessing a trait whose true prevalence was 50% (for which 50% is needed in the largest sample to reach a particular precision value).

### 2.5. Questionnaire

The questionnaire survey (Appendix A) for doctors consisted of 32 questions and was designed by the authors. It was preceded by preliminary information which consisted of an explanation of the purpose of the study, details on how to contact the author, and information about the voluntary and anonymous nature of the survey.

The survey consisted of both single-choice and multiple-choice questions. The age question was an open-ended question. The rest of the questions were closed questions. Nine questions related to knowledge about the HPV virus, eight related to the state of knowledge about the HPV vaccine. The rest of the questions were about the attitudes of doctors to vaccination and demographic data.

## 3. Results

### 3.1. Group Characteristics

The study included doctors during residency in pediatrics, gynecology, and obstetrics, and dermatology and venereology, participating in compulsory courses during their specialization studies. The questionnaires were completed by 639 doctors. A pooled analysis of doctors is presented in Table 1.

Altogether, 608 doctors answered the gender question, 492 women (80.9%) and 116 men (19.1%). Doctors 20–30 years of age accounted for 62.5% of the respondents (*n* = 362), doctors 30–40 years of age accounted for 34.4% (*n* = 199), and doctors 40–70 years of age accounted for 3.1% (*n* = 18). The largest group of doctors indicated town with over 500,000 inhabitants as their place of residence (45.4%; *n* = 275). A total of 204 doctors (33.7%) indicated town with 100,000 to 500,000 inhabitants as their place of residence, while 84 doctors (13.9%) indicated town with 20,000 to 100,000 inhabitants as their place of residence. Town up to 20,000 inhabitants was indicated by 25 doctors (4.1%). Inhabitants of villages accounted for 3% (*n* = 18).

Doctors specializing in dermatology and venereology constituted 31.8% (*n* = 203) of the subjects, in gynecology and obstetrics—32.1% (*n* = 205), in pediatrics—33.8% (*n* = 216). Almost all doctors (98.7%; *n* = 624) mentioned hospital as their place of employment. For most of them, it was the only place of employment—these doctors constituted 87.7% of the study group. Others combined employment in a hospital with employment in a clinic, private office, or other places of employment. Only 8 doctors (1.3%) did not work at a hospital. One of the respondents indicated three places of employment.

Further analyses were conducted taking into account the division of doctors into dermatology and venereology, gynecology and obstetrics, and pediatrics. The differences between the groups were statistically significant in terms of sex—in the group of doctors specializing in gynecology and obstetrics, the percentage of men was much higher than in the other two groups. There were also differences in the age of doctors—the youngest group was doctors specializing in dermatology and venereology. No major differences in the distribution of the place of residence and the place of employment were observed.

### 3.2. The State of Doctors’ Knowledge about HPV and Vaccination

The knowledge of doctors about HPV is low and the state of knowledge about HPV vaccination is very low in the study group.

Table 2 contains summary statements of the survey percentages. The summary is presented in two versions: within the limits set by quartiles which result in an uneven division; and within the limits selected after data analysis—below 50 and then every 10 points. When divided into quartiles, as many as 34.7% of doctors were in the lowest quartile in the range between 54–59% of correct answers, 21% of doctors achieved 60–65%, and 21% and 23.3% of doctors came in the two highest quartiles. The highest test result was 88% of correct answers and the lowest was 12% of correct answers.

Analyzing the second division, 21.4% (*n* = 137) of doctors obtained a result of up to 50% of correct answers in the range of 51–60% (*n* = 219), 61–70% (*n* = 134), and 71–100% (*n* = 149). The highest number of doctors obtained a score between 51% and 60% of correct answers. If this were a test, then as many as 55.7% would not pass the test, assuming that the pass mark was a score of more than 61% of correct answers.

Table 3 contains a summary list of components of answers to questions concerning doctors’ knowledge about the virus and HPV vaccinations. In this comparison, over 90% of the surveyed doctors answered six questions correctly (What does HPV stand for? Are all types of HPV highly oncogenic? Which types of HPV most often predispose to cervical cancer? Which types of HPV are the most common? Which cause genital warts? Does HPV vaccination protect 100% against cervical cancer?) and less than 10% of surveyed doctors gave correct answers to two questions (What types of virus does Cervarix protect against? How can you become infected by HPV?).

Table 4 presents a breakdown of the percentages of correct answers to questions about HPV and HPV vaccination. Almost half of the doctors (48.4%) had a score of less than 60% on the HPV questions. The state of knowledge about HPV vaccination was even lower: 76.6% of doctors obtained a result below 60%.

### 3.3. The State of Doctors’ Knowledge about HPV

Table 5 presents the percentage of correct answers given by doctors to questions about knowledge about HPV by specialization. There were no differences related to sex and age. It is worth noting that the largest number of doctors who gave incorrect answers to the question of “What HPV predisposes to” was pediatricians (74%) and this was statistically significant. Statistically significant differences are also visible in the responses to the methods of infection—the percentage of correct responses is also the lowest among pediatricians (it is also low for the other specializations). Risk factors for cervical cancer were correctly described by 43.8% of dermatologists and venereologists, and among gynecologists and obstetricians, and for pediatricians, this percentage was lower. When asked about the types of virus predisposing to cervical cancer and the incidence of condylomas, almost all dermatologists and venereologists answered correctly (95.6%), among the other two groups of doctors this percentage was lower (89.8% and 87.5%) and the differences were statistically significant.

### 3.4. The State of Doctors’ Knowledge about HPV Vaccines

Table 6 shows the percentage of correct answers given by doctors to questions about HPV vaccination divided by specialization. There were no major differences related to sex and age. There was a statistically significant higher percentage of correct answers by women to the question regarding the Silgard vaccine and a statistically significant higher percentage of correct answers regarding target group definition by the youngest age group.

There were more statistically significant differences in the correct vaccine answers between the specialization groups. Dermatologists and venereologists were more likely to answer correctly the question about the effectiveness of the vaccine, pediatricians responded best to questions about the number of doses and possible complications but identified the target group relatively worse.

In addition, the doctors’ answers to the question about the side effects of the vaccine deserve attention. In total, 352 doctors correctly identified vaccine-site pain and fainting as a scientifically proven side effect of the vaccine. However, as many as 59 doctors selected the answer “Anaphylactic reaction in children allergic to proteins, related to the cultivation of vaccine viruses on chicken embryos”. Moreover, 20 doctors selected the option “all of the above answers”, for a total of 22 doctors who believe that proven vaccine side effects include “Autism, ADHD (Attention deficit hyperactivity disorder), and other central nervous system disorders caused by thiomerosal, an ethylmercury compound used as a preservative in the vaccine”, and 79 doctors in total who believe that the vaccine may lead to “An anaphylactic reaction in children allergic to proteins connected with the cultivation of vaccine viruses in chicken embryos”. 

### 3.5. Doctors’ Attitudes towards Vaccination

Table 7 and Table 8 present the answers to the questions concerning the attitude to vaccination of the surveyed doctors, divided by age and specialization. No statistically significant differences were observed in the attitude of doctors to vaccinations in the division by gender. There was, however, an increasing trend with age in the percentage of doctors informing and persuading their patients to vaccinate their daughters against HPV. The distribution of knowledge in the persuading and non-vaccination groups is very similar. Among the considered specializations, it was statistically significant that doctors in the course of specialization in gynecology and obstetrics more often declared that they encourage their relatives to get vaccinated, that they inform their patients about vaccinations, that they encourage their daughters to get vaccinated, and that they would recommend vaccination if it were reimbursed. A few people (*n* = 20) directly said that they were not in favor of HPV vaccination, while 69 doctors indicated that they had no opinion. Among this group, more than half had a test result in the lowest quartile. Among doctors with a low-test result, 1.8% did not recommend HPV vaccination, and as many as 16.7% said that they had no opinion. In the group with the highest test result, 2% did not recommend HPV vaccination and 7.4% did not have an opinion.

### 3.6. Doctors’ Attitudes towards Vaccination by Test Result

Table 9 summarizes the questions on attitudes to vaccination by test score. There are significant differences between the groups designated by test result in the answers to questions regarding doctors’ declaration of supporting vaccination and the willingness to recommend the vaccine in the event of its reimbursement. There is a difference between the groups with the worst and best knowledge about vaccinations and HPV. Doctors whose test results were the highest are more likely to support vaccination and they would recommend vaccination more often if it were reimbursed. Differences in the perception of infection as a significant medical problem were on the verge of statistical significance. It can be seen that doctors with the lowest test result are less likely to perceive infection as a significant problem.

## 4. Discussion

The state of knowledge about HPV infections in Poland has only been assessed sporadically within various groups (medical students, nurses). Doctors were never included in those studies. Questionnaires were usually used to examine the state of knowledge of the above groups, but the study groups were not always well defined. Our study showed that the knowledge of doctors about HPV in the study group is low, independent of sex, age, and specialization.

The knowledge of doctors about the HPV vaccine in the study group is very low and independent of sex, age, and specialization. The state of doctors’ knowledge about HPV and the HPV vaccine has an impact on the attitude towards HPV vaccinations (recommending HPV vaccinations, recommending HPV vaccinations if they were reimbursed) and has no effect on pro-vaccine behaviors (encouraging or informing patients).

In international studies in the United States, the United Kingdom, and Australia, which included 2409 people, the percentage of people who had heard of HPV was 61%. This knowledge was influenced by education, marital status, having a daughter 9–17 years of age, and previous vaccination. The overall knowledge about HPV has been assessed as low, which may have a negative impact in countries where HPV testing is a screening test [12]. In other studies, the percentage of people who have heard of HPV fluctuated and averaged 77% among young women 19–26 years of age [13], and in a meta-analysis of 39 studies (19,986 people)—between 13% and 93% [14].

Previous studies of the state of knowledge among health professionals have shown a high general knowledge about HPV, and the highest results are among doctors who are women [15]. However, the state of knowledge varies between doctors of various specialties, private and public health care workers, and between doctors who treat less than 15 patients a day and doctors who treat more than 15 patients a day [16]. This is reflected in the presented results of this study, which show statistically significant differences in the assessment of the state of knowledge among doctors of various specializations.

The HPV vaccine is registered in Poland for young girls who are sexually inactive. Among doctors who took part in the study, pediatricians will most often have contact with this group. It is even more disturbing that the lowest test result in the field of knowledge about the HPV virus was obtained by doctors who specialize in pediatrics. A few studies conducted in the world on groups of pediatricians confirm their low level of knowledge about HPV [17].

In the study conducted on a group of doctors, knowledge about the HPV vaccine was lower than the knowledge about HPV. Among the three study groups, the lowest score in terms of knowledge about the HPV vaccine was obtained by doctors who specialize in dermatology and venereology. Due to the fact that dermatologists and venereologists very often diagnose and treat patients with genital warts, pre-cancerous conditions of the external genital area, e.g., vulvar neoplasia, penile neoplasia, and peri-anal neoplasia. Training in dermatology and venereology also covers the prevention of HPV infections with the use of vaccines against these viruses. In the specialization program, knowledge of this subject is obligatory. Dermatologists should know the spectrum of protection of HPV infections depending on the type of vaccine. There are no other studies in this research field in the literature, apart from a single study that does not describe in detail the state of knowledge of dermatologists [18]. In a study by Warner et al. in which the analysis included family doctors, pediatricians, and nurses, a low level of knowledge about the vaccine among pediatricians was noted [16]. In addition, it was found that the state of knowledge depended on the age of the respondent, the place of employment (a higher level of knowledge among university employees), and the number of daily patients (higher among doctors seeing 20–29 patients a day). 

In the results of this study, the state of doctors’ knowledge about the HPV virus and the HPV vaccine has an impact on the attitude towards HPV vaccinations (recommending HPV vaccinations, recommending HPV vaccinations if it were reimbursed), and it does not affect pro-vaccination behavior (persuading, informing patients). Only one doctor among the study group cited the fear that the vaccine might induce sexual behavior in children as a reason for being anti-HPV (results not included).

The study by Rosenthal et al. also noted that women who were strongly persuaded to vaccinate were four times more likely to get vaccinated than those who were poorly persuaded [19]. Doctors were encouraged to vaccinate patients depending on their knowledge, views, and values. The following barriers were mentioned that limited doctors from persuading patients to vaccinate: lack of time, the need to fill in additional medical documentation, concerns about the low effectiveness and safety of the vaccine, the avoidance of follow-up by vaccinated girls, and the belief that the vaccine encourages sexual promiscuity [20].

There may be some possible limitations in this study. Our research takes into consideration only one aspect of the barriers towards vaccination, namely: doctors’ knowledge. Further investigations need to be conducted to include more anti-vaccine factors.

## 5. Conclusions

Based on the research, the following conclusions were drawn. The knowledge of doctors about HPV in the study group is low, independent of sex, age, and specialization. The knowledge of doctors about the HPV vaccine in the study group is very low and independent of sex, age, and specialization. The state of doctors’ knowledge about the HPV virus and HPV vaccine has an impact on the attitude towards HPV vaccinations (recommending HPV vaccinations, recommending HPV vaccinations if it were reimbursed), and it does not affect pro-vaccination behavior (encouraging, informing patients).

This research is the first attempt in Poland to identify the most important barriers to the effective implementation of the HPV vaccine and, therefore, to the prevention of diseases associated with this virus. The presented analysis may help in the implementation of HPV vaccination programs in Poland.

## Figures and Tables

**Table 1:** Characteristic of the group.

Characteristic	Group Size	Options	*N* (%)
Sex	608	Woman	492 (80.9%)
		Men	116 (19.1%)
Age group	579	20–30 years	362 (62.5%)
		30–40 years	199 (34.4%)
		40–70 years	18 (3.1%)
Place of residency	606	Countryside	18 (3%)
		town up to 20,000 inhabitants	25 (4.1%)
		town from 20,000 to 100,000 inhabitants	84 (13.9%)
		town from 100,000 to 500,000 inhabitants	204 (33.7%)
		town > 500,000 inhabitants	275 (45.4%)
Place of employment	632	Other	3 (0.5%)
		Outpatient Clinic	5 (0.8%)
		Hospital	554 (87.7%)
		Hospital, Private practice	13 (2.1%)
		Hospital, other	4 (0.6%)
		Hospital, Outpatient Clinic	34 (5.4%)
		Hospital, Outpatient Clinic, Private Practice	18 (2.8%)
		Hospital, Outpatient Clinic, Private Practice, other	1 (0.2%)
Specialization	639	Dermatology and Venereology	203 (31.8%)
		Dermatology and Venereology, other	1 (0.2%)
		Gynecology and Obstetrics	205 (32.1%)
		Other	12 (1.9%)
		Family Medicine, Pediatrics	1 (0.2%)
		Pediatrics	216 (33.8%)

**Table 2:** Summary test results.

Variable	Test Result	*n* (%)
Percentage division *n* (%)	12–53%	222 (34.7%)
	53–59%	134 (21%)
	59–65%	134 (21%)
	65–88%	149 (23.3%)
Percentage division 1 *n* (%)	0–50%	137 (21.4%)
	50–60%	219 (34.3%)
	60–70%	134 (21%)
	70–100%	149 (23.3%)

**Table 3:** Knowledge about HPV vaccination and the HPV virus—correct answers to component questions.

Question	*n* (%)
Q4 Are all types of HPV highly oncogenic?	623 (97.5%)
Q5 HPV infection predisposes to:	284 (44.4%)
Q6 How can somebody get infected with HPV?	53 (8.3%)
Q7 How can HPV infection be prevented, or the risk of HPV infection be reduced?	508 (79.5%)
Q8 What factors increase the risk of developing cervical cancer?	233 (36.5%)
Q9 What percentage of head and neck cancers (cancer of the mouth, tonsils, upper throat) in Poland are related to HPV infection?	280 (43.8%)
Q10 Which types of HPV most often predispose to cervical cancer?	581 (90.9%)
Q11 Which types of HPV are most likely to cause genital warts?	581 (90.9%)
Q12 What does the HPV vaccine contain?	336 (52.6%)
Q14 Does HPV vaccination give 100 percent protection against cervical cancer?	600 (93.9%)
Q15 What types of viruses does Silgard give protection against?	432 (67.6%)
Q16 What types of viruses does Cervarix give protection against?	12 (1.9%)
Q17 How many doses of the HPV vaccine should be administered?	266 (41.6%)
Q18 The target groups for the vaccine are:	132 (20.7%)
Q20 Is the cost of the vaccine in Poland reimbursed?	509 (79.7%)
Q21 The scientifically proven complications of HPV vaccination include:	352 (55.1%)

**Table 4:** Summary test results of HPV knowledge and vaccine knowledge.

Test	% Points	*N*	%
Knowledge (HPV)	10	1	0.2%
	20	3	0.5%
	30	10	1.6%
	40	45	7.0%
	50	91	14.2%
	60	159	24.9%
	70	190	29.7%
	80	110	17.2%
	90	29	4.5%
	100	1	0.2%
	0–50	150	23.5%
	50–60	159	24.9%
	60–70	190	29.7%
	70–100	140	21.9%
Knowledge (vaccine)	0	4	0.6%
	14	18	2.8%
	29	86	13.5%
	43	176	27.5%
	57	209	32.7%
	71	127	19.9%
	86	19	3.0%
	0–30	104	16.3%
	30–60	385	60.3%
	60–100	146	22.8%

**Table 5:** Correct answers to the questions about HPV depending on the specialization.

Question	Dermatology and Venereology (*n* = 203)	Gynecology and Obstetrics (*n* = 205)	Pediatrics (*n* = 216)	*p*
Q3 What does HPV stand for?	198 (97.5%)	203 (99.0%)	212 (98.1%)	0.517
Q4 Are all types of HPV highly oncogenic?	200 (98.5%)	201 (98.0%)	207 (95.8%)	0.175
Q5 HPV infection predisposes to:	115 (56.7%)	108 (52.7%)	55 (25.5%)	<0.001
Q6 How can somebody get infected with HPV?	20 (9.9%)	24 (11.7%)	8 (3.7%)	0.008
Q7 How can HPV infection be prevented, or the risk of HPV infection be reduced?	170 (83.7%)	164 (80.0%)	163 (75.5%)	0.108
Q8 What factors increase the risk of developing cervical cancer?	89 (43.8%)	70 (34.1%)	68 (31.5%)	0.023
Q9 What percentage of head and neck cancers (cancer of the mouth, tonsils, upper throat) in Poland are related to HPV infection?	86 (42.4%)	91 (44.4%)	97 (44.9%)	0.859
Q10 Which types of HPV most often predispose to cervical cancer?	195 (96.1%)	179 (87.3%)	194 (89.8%)	0.006
Q11 Which types of HPV are most likely to cause genital warts?	194 (95.6%)	184 (89.8%)	189 (87.5%)	0.013

**Table 6:** Correct answers to the questions about HPV vaccination depending on the specialization.

Question	Dermatology and Venereology (*n* = 203)	Gynecology and Obstetrics (*n* = 205)	Pediatrics (*n* = 216)	*p*
Q12 What does the HPV vaccine contain?	114 (56.2%)	114 (55.6%)	102 (47.2%)	0.119
Q14 Does HPV vaccination give 100 percent protection against cervical cancer?	199 (98.0%)	192 (93.7%)	195 (90.3%)	0.004
Q15 What types of viruses does Silgard give protection against?	130 (64.0%)	145 (70.7%)	147 (68.1%)	0.347
Q16 What types of viruses does Cervarix give protection against?	2 (1.0%)	2 (1.0%)	6 (2.8%)	0.235
Q17 How many doses of the HPV vaccine should be administered?	60 (29.6%)	78 (38.0%)	121 (56.0%)	<0.001
Q18 The target groups for the vaccine are:	47 (23.2%)	51 (24.9%)	32 (14.8%)	0.024
Q20 Is the cost of the vaccine in Poland reimbursed?	156 (76.8%)	170 (82.9%)	170 (78.7%)	0.296
Q21 The scientifically proven complications of HPV vaccination include:	96 (47.3%)	104 (50.7%)	147 (68.1%)	<0.001

**Table 7:** Doctors’ attitude to HPV vaccinations depending on age.

Question	Options	20–30% (*n* = 362)	30–40% (*n* = 199)	40–70% (*n* = 18)	No Answer	*p*
Q22 Encourages relatives (family/friends) to vaccinate their daughters against HPV?	Yes	235 (66.4%)	142 (72.4%)	13 (72.2%)	13 (2.0%)	0.322
Q24 Informs patients about the possibility of vaccinating their daughters against HPV	Yes	177 (49.7%)	123 (62.4%)	15 (83.3%)	10 (1.6%)	0.001
Q25 Encourages patients to vaccinate their daughters against HPV	Yes	163 (45.4%)	109 (54.8%)	13 (72.2%)	6 (0.9%)	0.015
Q26 Considers HPV infection to be a significant medical problem	Yes	339 (93.9%)	181 (91.0%)	17 (94.4%)	2 (0.3%)	0.247
	No	4 (1.1%)	5 (2.5%)	1 (5.6%)		
	No opinion	18 (5.0%)	13 (6.5%)	0 (0.0%)		
Q27 Is a supporter of HPV vaccination	Yes	317 (87.6%)	171 (85.9%)	15 (83.3%)	1 (0.2%)	0.648
	No	12 (3.3%)	5 (2.5%)	1 (5.6%)		
	No opinion	33 (9.1%)	23 (11.6%)	2 (11.1%)		
Q29 Thinks that the vaccine should be reimbursed in Poland	Yes	321 (88.7%)	171 (88.1%)	16 (88.9%)	36 (5.6%)	0.750
	No	13 (3.6%)	10 (5.2%)	0 (0.0%)		
	No opinion	28 (7.7%)	13 (6.7%)	2 (11.1%)		
Q30 Would recommend HPV vaccine if it were reimbursed	Yes	327 (90.3%)	175 (88.8%)	14 (82.4%)	34 (5.3%)	0.461
	No	8 (2.2%)	7 (3.6%)	1 (5.9%)		
	I do not know	27 (7.5%)	15 (7.6%)	2 (11.8%)		

**Table 8:** Doctors’ attitude to HPV vaccinations depending on specialization.

Question	Options	Dermatology and Venereology (*n* = 203)	Gynecology and Obstetrics (*n* = 205)	Pediatrics (*n* = 216)	No Answer	*p*
Q22 Encourages relatives (family/friends) to vaccinate their daughters against HPV?	Yes	133 (66.5%)	153 (75.4%)	134 (64.1%)	13 (2.0%)	0.035
Q24 Informs patients about the possibility of vaccinating their daughters against HPV	Yes	100 (50.3%)	133 (65.5%)	109 (51.2%)	10 (1.6%)	0.002
Q25 Encourages patients to vaccinate their daughters against HPV	Yes	88 (43.8%)	123 (60.0%)	95 (44.6%)	6 (0.9%)	0.001
Q26 Considers HPV infection to be a significant medical problem	Yes	187 (92.6%)	194 (94.6%)	195 (90.7%)	2 (0.3%)	0.370
	No	4 (2.0%)	4 (2.0%)	3 (1.4%)		
	No opinion	11 (5.4%)	7 (3.4%)	17 (7.9%)		
Q27 Is a supporter of HPV vaccination	Yes	176 (87.1%)	181 (88.3%)	180 (83.3%)	1 (0.2%)	0.123
	No	7 (3.5%)	8 (3.9%)	4 (1.9%)		
	No opinion	19 (9.4%)	16 (7.8%)	32 (14.8%)		
Q29 Thinks that the vaccine should be reimbursed in Poland	Yes	166 (88.8%)	175 (89.3%)	180 (87.8%)	36 (5.6%)	0.713
	No	5 (2.7%)	9 (4.6%)	7 (3.4%)		
	No opinion	16 (8.6%)	12 (6.1%)	18 (8.8%)		
Q30 Would recommend HPV vaccine if it were reimbursed	Yes	160 (86.0%)	182 (91.9%)	187 (90.8%)	34 (5.3%)	0.029
	No	4 (2.2%)	8 (4.0%)	3 (1.5%)		
	No answer	22 (11.8%)	8 (4.0%)	16 (7.8%)		

**Table 9:** Doctors’ attitude to HPV vaccinations depending on test result.

Question	Options	*n*	0–50%	*n*	50–60%	*n*	60–70%	*n*	70–88%	*p*
Q22 Encourages relatives (family/friends) to vaccinate their daughters against HPV?	Yes	132	80 (60.6%)	213	153 (71.8%)	134	90 (67.2%)	147	105 (71.4%)	0.134
	No		52 (39.4%)		60 (28.2%)		44 (32.8%)		42 (28.6%)	
Q24 Informs patients about the possibility of vaccinating their daughters against HPV	Yes	133	70 (52.6%)	214	121 (56.5%)	134	74 (55.2%)	148	84 (56.8%)	0.888
	No		63 (47.4%)		93 (43.5%)		60 (44.8%)		64 (43.2%)	
Q25 Encourages patients to vaccinate their daughters against HPV	Yes	135	61 (45.2%)	216	114 (52.8%)	134	59 (44.0%)	148	77 (52.0%)	0.274
	No		74 (54.8%)		102 (47.2%)		75 (56.0%)		71 (48.0%)	
Q26 Considers HPV infection to be a significant medical problem	Yes	136	117 (86.0%)	218	204 (93.6%)	134	129 (96.3%)	149	140 (94.0%)	0.059
	No		5 (3.7%)		3 (1.4%)		1 (0.7%)		2 (1.3%)	
	No opinion		14 (10.3%)		11 (5.0%)		4 (3.0%)		7 (4.7%)	
Q27 Is a supporter of HPV vaccination	Yes	136	108 (79.4%)	219	193 (88.1%)	134	113 (84.3%)	149	135 (90.6%)	<0.001
	No		0 (0.0%)		10 (4.6%)		7 (5.2%)		3 (2.0%)	
	No opinion		28 (20.6%)		16 (7.3%)		14 (10.4%)		11 (7.4%)	
Q29 Thinks that the vaccine should be reimbursed in Poland	Yes	127	106 (83.5%)	207	185 (89.4%)	129	112 (86.8%)	140	130 (92.9%)	0.276
	No		8 (6.3%)		7 (3.4%)		4 (3.1%)		4 (2.9%)	
	No opinion		13 (10.2%)		15 (7.2%)		13 (10.1%)		6 (4.3%)	
Q30 Would recommend HPV vaccine if it were reimbursed	Yes	128	111 (86.7%)	206	184 (89.3%)	130	112 (86.2%)	141	135 (95.7%)	0.043
	No		2 (1.6%)		6 (2.9%)		4 (3.1%)		4 (2.8%)	
	I do not know		15 (11.7%)		16 (7.8%)		14 (10.8%)		2 (1.4%)	

## Abbreviations

HPV	human papillomavirus
RRP	recurrent respiratory papillomatosis
CIN	cervical intraepithelial neoplasia
VIN	vulval intraepithelial neoplasia
VaIN	vaginal intraepithelial neoplasia
AIN	anal intraepithelial neoplasia
PIN	penile intraepithelial neoplasia
NHP	National Health Provider
NFZ	Narodowy Fundusz Zdrowia
STD	sexually transmitted disease
ADHD	Attention deficit hyperactivity disorder

## Appendix A. Questionnaire

**1. What is your specialization:**

*You can tick several options*

◻	Gynecology and obstetrics
◻	Family Medicine
◻	Dermatology and Venereology
◻	Urology
◻	Pediatrics
◻	Other: _ _ _ _ _ _ _ _ _ _ _ _ _ _ _ _ _ _ _ _ _ _ _ _ _ _ _ _

**2. Where is your main place of employment?**

*You can tick several options*

◻	Hospital
◻	Outpatient Clinic
◻	Private Practice
◻	Other: _ _ _ _ _ _ _ _ _ _ _ _ _ _ _ _ _ _ _ _ _ _ _ _ _ _ _ _

**3. What does the HPV stand for?**

*Select one answer*

◻	Cytomegalovirus
◻	Human papillomavirus
◻	Herpes simplex virus
◻	I do not know

**4. Are all types of HPV highly oncogenic?**

*Select one answer*

◻	Yes
◻	No
◻	I do not know

**5. HPV infection predisposes to:**

*You can tick several options*

◻	Cancer of the genitourinary organs (vagina, penis, anus, vulva)
◻	Cervical cancer
◻	Head and neck cancer
◻	Papillary lesions of the genital area
◻	Respiratory papillomatosis
◻	I do not know

**6. How can somebody get infected with HPV?**

*You can tick several options*

◻	Through a kiss
◻	By touch
◻	By sexual intercourse
◻	During natural childbirth
◻	By contact of infected blood with the blood of an uninfected person, e.g., using the same needle
◻	I do not know

**7. How can HPV infection be prevented, or the risk of HPV infection be reduced?**

*You can tick several options*

◻	By vaccination before sexual initiation
◻	By using condoms
◻	By limiting the number of sexual partners and by avoiding risky sexual behavior
◻	It is not possible to prevent HPV infection
◻	I do not know

**8. What factors increase the risk of developing cervical cancer?**

*You can tick several options*

◻	Smoking
◻	A family history of cervical cancer
◻	HPV infection
◻	A large number of sexual partners
◻	Lack of physical activity
◻	I do not know

**9. What percentage of head and neck cancers (cancer of the mouth, tonsils, upper throat) in Poland are related to HPV infection?**

*Select one answer*

◻	Up to around 30%
◻	30–50%
◻	Above 50%
◻	I do not know

**10. Which types of HPV most often predispose to cervical cancer?**

*You can tick several options*

◻	51 and 52
◻	1 and 2
◻	16 and 18
◻	45 and 47
◻	I do not know

**11. Which types of HPV are most likely to cause genital warts?**

*You can tick several options*

◻	6 and 11
◻	7 and 15
◻	89 and 90
◻	1 and 2
◻	I do not know

**12. What does the HPV vaccine contain?**

*Select one answer*

◻	A small number of live viruses to stimulate an immune response
◻	HPV Antibodies
◻	HPV envelope devoid of DNA
◻	Virus-like particles with damaged DNA
◻	I do not know

**13. What type of vaccine is most often used in Poland?**

*Select one answer*

◻	Monovalent
◻	Bivalent
◻	Quadrivalent
◻	I do not know

**14. Does HPV vaccination give 100 percent protection against cervical cancer?**

*Select one answer*

◻	Yes
◻	No
◻	I do not know

**15. What types of viruses does Silgard give protection against?**

*Select one answer*

◻	6, 11, 16, and 18
◻	16 and 18
◻	6 and 11
◻	I do not know

**16. What types of viruses does Cervarix give protection against?**

*Select one answer*

◻	6, 11, 16, and 18
◻	16 and 18
◻	6 and 11
◻	I do not know

**17. How many doses of the HPV vaccine should be administered?**

*Select one answer*

◻	Always 2 doses regardless of the type of vaccine
◻	2 or 3 doses depending on the type of vaccine and the age of the patient
◻	Always 3 doses regardless of the type of vaccine
◻	4 doses for Silgard or 1 dose for Cervarix
◻	I do not know

**18. The target groups for the vaccine are:**

*You can tick several options*

◻	Young women / girls around 12 years old
◻	Young men / boys around 12 years old
◻	Young women before sexual initiation
◻	Young men before sexual initiation
◻	Young women not infected with HPV
◻	Young men not infected with HPV
◻	All women, regardless of age
◻	All men, regardless of age
◻	I do not know

**19. Is it possible to get vaccinated against HPV at your place of employment?**

*Select one answer*

◻	Yes
◻	No
◻	I do not know

**20. Is the cost of the vaccine in Poland reimbursed?**

*Select one answer*

◻	Yes, 100%
◻	Yes, 50%
◻	Yes, but I do not know how much is reimbursed
◻	No
◻	I do not know

**21. The scientifically proven complications of HPV vaccination include:**

*Select one answer*

◻	Pain at the site of vaccination and fainting after vaccination
◻	Autism, ADHD and other central nervous system disorders caused by thiomersal (an ethylmercury compound used as a preservative in the vaccine)
◻	An anaphylactic reaction in children allergic to proteins connected with the cultivation of vaccine viruses in chicken embryos
◻	All of the above
◻	None of the above

**22. Do you encourage your relatives (family/friends) to vaccinate their daughters against HPV?**

*Select one answer*

◻	Yes
◻	No

**23. How much time do you spend talking about disease prophylaxis during a patient’s visit or stay in your ward?**

*Select one answer*

◻	1 minute
◻	5 minutes
◻	More than 5 minutes
◻	I do not have time for these conversations

**24. Do you inform your patients about the possibility of vaccinating their daughters against HPV?**

*Select one answer*

◻	Yes
◻	No

**25. Do you encourage your patients to vaccinate their daughters against HPV?**

*Select one answer*

◻	Yes
◻	No

**26. Do you consider HPV infection to be a significant medical problem?**

*Select one answer*

◻	Yes
◻	No
◻	I have no opinion

**27. Do you support HPV vaccination?**

*Select one answer*

◻	Yes
◻	No
◻	I have no opinion

**28. If you selected NO to question number 27, why do you not support HPV vaccination?**

*Select one answer*

◻	The high price of the vaccine
◻	The side effects of the vaccine
◻	The fear that the vaccine may encourage children to engage in risky sexual behaviors
◻	The reluctance to educate children about human sexuality
◻	I think that this vaccine is unnecessary
◻	I think that this vaccine is ineffective
◻	I think that this vaccine is dangerous to health
◻	Other: _ _ _ _ _ _ _ _ _ _ _ _ _ _ _ _ _ _ _ _ _ _ _ _ _ _ _ _

**29. Do you think that the vaccine should be reimbursed in Poland?**

*Select one answer*

◻	Yes
◻	No
◻	I have no opinion

**30. Would you recommend your patients to vaccinate against HPV if it were reimbursed?**

*Select one answer*

◻	Yes
◻	No
◻	I have no opinion

**31. How old are you?**

……………………..

**32. Please select your gender:**

◻	Female
◻	Male
